# Medical News

**Published:** 1854-01

**Authors:** 


### MEDICAL NEWS.

We are happy to see, by our foreign journals, that the Cholera is on the decline throughout all Europe.

An excellent regulation prevails in many parts of England, having for its object the checking of the spread of this disease. We allude to the appointment of physicians as sanitary Inspectors of their several districts. Their duties are to report nuisances of all kinds to the local boards of health and to visit, in case of death, the house in which it took place,and administer prompt medical relief to every case of diarrhœa. This latter provision for the poor, who are often both negligent and apathetic, is considered necessary, as there is a very general belief that this premonitory condition exists in every instance.

Another interesting fact seems to have been established; which is, that the same localities, even the same houses, have been visited by this disease, in its several epidemics.

We mention these matters, as they have an important bearing upon the direction, which should be given to our preventive and sanitary efforts, should we again have the misfortune to be visited by this pestilence.

---

Two cases of nearly fatal accidents, caused in each instance by the inhalation of a mixture composed of two drachms of chloroform and four of ether, are reported by Dr. W. H. Mussey, of Cincinnati. Both patients were apparently dead; there being in one an entire suspension of the action of the heart and lungs for upwards of five minutes. Reanimation was induced in both by artificial respiration and irritation of the glottis.

We have no doubt that such casualties occur much more frequently than is supposed. Chloroform is occasionally so unmanageable as to astonish even its friends, and we should not be surprised if, after destroying a few more lives, and occasioning a few more suits, some other less dangerous anæsthetic should entirely take its place.

---

During the month of November, 1141 deaths from cholera occurred on board of emigrant ships bound from Europe to New York, and between four and five thousand were afflicted with it during the passage. That this awful mortality was mainly due to filth and ill-ventilation is shown by the fact, that in some of the vessels where exercise upon deck was enforced with the passengers, and the steerage cleansed and ventilated, not a single death took place.

Fourteen thousand dollars were recently presented to Harvard College by Dr. Shattuck, of Boston, for the purpose of founding a chair of Morbid Anatomy. It will be called the "Shattuck Professorship of Morbid Anatomy."

---

Dr. Levin S. Joynes, of Accomac, was appointed at the late meeting of the Society of Alumni of the University of Virginia, to deliver the next annual address before that body.

---

Dr. Socrates Maupin has been elected Professor of Chemistry in the University of Virginia, to fill the vacancy caused by the resignation of Dr. J. Lawrence Smith.

---

Dr. R. J. Breckenridge has been elected to the chair of Materia Medica, in the Kentucky School of Medicine, at Louisville, in place of Dr. E. D. Force, resigned.

---

Dr. H. L. Thomas has been compelled by the pressure of other duties, to withdraw from the editorial charge of the Virginia Medical and Surgical Journal. He will continue, however, to be a contributor to its pages. Dr. Otis has now the sole charge of the Journal.

---

We regret to announce the death of Dr. Hester, of New Orleans. Dr. Hester was widely and favorably known to the profession as the Editor of the New Orleans Medical and Surgical Journal.

---

We have been unable to notice this year's "Transactions of the American Medical Association," having been disappointed, though so near head-quarters, in receiving a copy.

---

At the semi-annual meeting (Oct. 26th) of the Medical Society of Virginia, at Charlottesville, Dr. T. P. Atkinson in the Chair, and twenty five delegates present, the following scheme for the publication of a Medical Journal, (embodied in a report,) was recommended and finally adopted. "That the Journal be published either monthly or quarterly, as shall be deemed best by the executive committee. That it be conducted by a corps of editors, to be chosen annually by the Society, and all vacancies which may occur during the intervals between the meeting, to be filled by the executive committee. This corps to consist of seven individuals, one of whom shall be the publishing and financial agent, who only shall be paid for his services. To each of the other six a separate department shall be assigned, and no article shall appear which is not approved by a majority of the whole corps."

"To insure the services of a competent individual, whose whole time and energies shall be devoted to this work, we recommend that his salary should not be less than $1.500, (amended to $800,) nor more than $2000."

In the report on "Medical Education and Remedy for defects in it," presented and adopted at the same meeting, is incorporated the following. "We, therefore, suggest that the Medical Society of Virginia respectfully urge upon the Legislature, through the medium of a committee, the propriety of founding in the city of Richmond, a National (State) Medical School, under the control of a board of visitors; which board of visitors shall consist,—1st. Of the five members of the Supreme Court of Appeals. 2d. That the president of the Medical Society of Virginia shall, ex-officio, be a member of the board. 3d. That the president and directors of the literary fund shall appoint a member of that board."

The following resolution was adopted *unanimously*. "Resolved, that this society now reiterates the resolves and declarations heretofore made by it, on the subject of medical education and medical reform; that it especially reiterates its recommendations to the Legislature to pass the law for the establishment of a licensing board of medical examiners, which has been, and will be again brought to the consideration of the Legislature."

Dr. J. P. Atkinson has been appointed the financial editor of the Journal, which is to be issued under the auspices of the State Society: the assistant editors are Drs. Bolton, Lewis, Cabell, Thweat, Marx, and C. Johnson.

We cannot, under any aspect, see the necessity of this movement, Virginia having *already* two well established Journals, the Stethoscope and the Medical and Surgical Journal, both of which are admirably conducted and edited.

---

Notice.—We have been requested to publish the following:

The seventh annual meeting of the American Medical Association will be held in the city of St. Louis, on Tuesday, May 2d, 1854.

The secretaries of all societies and all other bodies entitled to representation in the Association are requested to forward to the undersigned correct lists of their respective delegations *as soon as they may be appointed;* and it is *earnestly* desired by the committee of arrangements that the appointments be made at as early a period as possible.

The following are extracts from Art. 2d of the Constitution:

"Each local society shall have the privilege of sending to the Association one delegate for every ten of its regular resident members, and one for every additional fraction of more than half of this number. The faculty of every regularly constituted medical college or chartered school of medicine shall have the privilege of sending two delegates. The professional staff of every chartered or municipal hospital containing a hundred inmates or more, shall have the privilege of sending two delegates; and every other permanently organized medical institution of good standing shall have the privilege of sending one delegate."

"Delegates representing the medical staffs of the United States Army and Navy shall be appointed by the chiefs of the army and navy medical bureaux. The number of delegates so appointed shall be four from the army medical officers, and an equal number from the navy medical officers."

The latter clause, in relation to delegates from the army and navy, was adopted as an amendment to the Constitution at the last annual meeting of the Association held in New York, in May, 1853.

E. S. Lemoine,
One of the Secretaries, St. Louis.

The Medical Press of the United States is respectfully requested to copy the foregoing.

